# Reduced handgrip strength and body mass index, gender, and diagnosis are associated with length of stay in hospitalized patients

**DOI:** 10.1590/0102-67202026000005e1934

**Published:** 2026-06-22

**Authors:** Lucas Rosasco MAZZINI, Vania Aparecida LEANDRO-MERHI

**Affiliations:** 1 Pontifícia Universidade Católica de Campinas, Faculty of Medicine – Campinas (SP), Brazil.; 2 Pontifícia Universidade Católica de Campinas, Postgraduate Program in Health Sciences – Campinas (SP), Brazil.

**Keywords:** Muscle strength, Nutritional status, Disease, Length of stay, Força muscular, Estado nutricional, doença, Tempo de internação

## Abstract

**Background:**

Handgrip strength assessment may be a useful indicator for nutritional diagnosis and association with outcomes.

**Aims:**

To investigate the relationship between handgrip strength and clinical outcomes in hospitalized patients.

**Methods:**

A cross-sectional study with 100 patients, investigating clinical outcome variables and explanatory variables (disease, surgery, comorbidities, gender, age, handgrip strength, body mass index, and subjective global assessment). The χ^2^ test, Fisher’s exact test, Mann-Whitney test, Spearman correlation coefficient, simple and multiple Cox regression analysis, and the Poisson regression model were used for data analysis.

**Results:**

The variables that, together, best explained the length of hospital stay were handgrip strength (p<0.0001; estimated parameter=-0.020432; standard error=0.003443), body mass index (p<0.0001; estimated parameter=-0.029020; standard error=0.004952), gender (p<0.0001; estimated parameter=0.302456; standard error=0.069615), and diagnosis. Body mass index and handgrip strength were inversely associated with the length of hospital stay. Male gender and vascular or orthopedic disease, compared to other diseases, were also associated with a higher length of hospital stay. No variable was considered an associated factor for complications.

**Conclusions:**

Handgrip strength and body mass index were inversely associated with the length of hospital stay. Male gender and vascular or orthopedic disease, compared to other diseases, were associated with a higher length of hospital stay.

## ARTICLE HIGHLIGHTS

A higher mean (or median) age, lower mean handgrip strength, and a higher rate of vascular disease, lung disease, and neoplasms were observed in patients with at least one comorbidity.

Body mass index and mean handgrip strength were inversely associated with the length of hospital stay (the lower the values, the longer the hospital stay).

Male gender and vascular or orthopedic disease, compared to other diseases, evidenced longer hospital stays.

## Summary

**CENTRAL MESSAGE:**

The association between patients’ malnutrition in the hospital setting and unfavorable clinical outcomes has already been well established in the literature. The loss of muscle mass is a critical malnutrition finding observed in patients with acute and chronic diseases. An alternative method to estimate muscle mass that could contribute as a relevant indicator for assessing the nutritional status of hospitalized patients is the assessment of handgrip strength, as a marker of health status in older adults.

**PERSPECTIVES:**

Handgrip strength and body mass index were inversely associated with the length of hospital stay. Male gender and vascular or orthopedic disease, compared to other diseases, were associated with a longer hospital stay.

## VISUAL ABSTRACT

**Figure:**
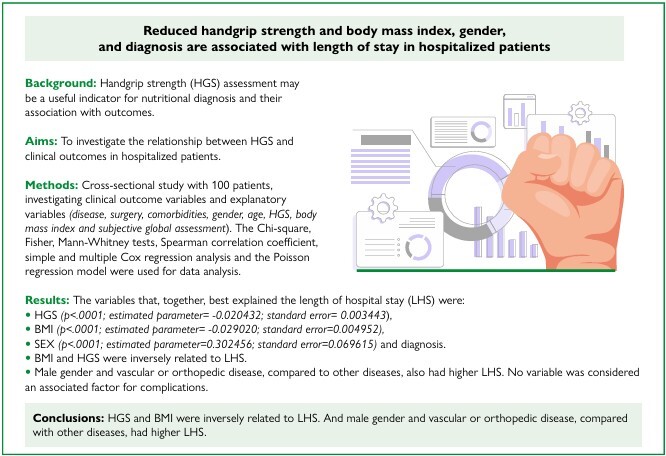

## RESUMO

**Racional:**

A avaliação da força de preensão palmar (FPP) pode ser um indicador útil para o diagnóstico nutricional e associação com desfechos.

**Objetivos:**

Investigar a relação entre a FPP e desfechos clínicos em pacientes hospitalizados.

**Métodos:**

Estudo transversal com 100 pacientes, sendo investigado variáveis de desfechos clínicos e variáveis explanatórias (doença, cirurgia, comorbidades, sexo, idade, FPP, índice de massa corporal e avaliação subjetiva global). Para análise dos dados foram utilizados os testes χ^2^, Fisher, Mann-Whitney, coeficiente de correlação de Spearman, análise de regressão de Cox simples e múltipla e o modelo de regressão de Poisson.

**Resultados:**

As variáveis que, em conjunto, melhor explicaram o tempo de internação (TI) foram a FPP (p<0,0001; parâmetro estimado=-0.020432; erro padrão=0.003443), IMC (p<0,0001; parâmetro estimado=-0.029020; erro padrão=0.004952), sexo (p<0,0001; parâmetro estimado=0.302456; erro padrão=0.069615) e diagnóstico. O IMC e a FPP foram inversamente relacionados ao TI. O sexo masculino e doença vascular ou ortopédica, comparadas a outras doenças, também apresentaram maior TI. Nenhuma variável foi considerada fator associado para complicações.

**Conclusões:**

A FPP e o IMC foram inversamente relacionados ao TI. E o sexo masculino e doença vascular ou ortopédica, comparadas com outras doenças, apresentaram maior TI.

## INTRODUCTION

 The association between patients’ malnutrition in the hospital setting and unfavorable clinical outcomes has already been well established in the literature^6,15,23^. When it comes to the nutritional diagnosis of inpatients, several tools and indicators are routinely used in hospital practice to identify malnutrition or nutritional risk^14,21,25,29,43^. The loss of muscle mass^12^ is a critical malnutrition finding observed in patients with acute and chronic diseases^32,33^ and in hospitalized elderly patients^41^. 

 An alternative method to estimate muscle mass that could contribute as a relevant indicator for assessing the nutritional status of hospitalized patients is the assessment of handgrip strength (HGS)^3,31,36^. HGS has been proposed as a marker of health status in older adults^4^. Some studies have shown an association between reduced HGS in adults and an increased risk of functional limitations and disability at older ages^31^. Because muscle function is affected early by nutritional deprivation, HGS has been considered a malnutrition marker^31^. 

 It has also been observed that HGS was the most sensitive method for identifying inpatients at risk of malnutrition, compared to anthropometric indicators^27^. Thus, this tool could be used as an alternative measure for detecting malnutrition^27^ and also for assessing muscle mass and managing malnutrition in hospitalized elderly patients^1^. The predictive validity of HGS for the length of hospital stay is also noteworthy, as observed in a retrospective study of patients undergoing abdominal surgery. Regression models showed that HGS was significantly and inversely associated with the length of hospital stay^24^. Thus, HGS could be considered a reliable clinical method for nutritional assessment in different clinical situations^2,19,24^ and a good indicator of overall muscle strength, as described in the revised European consensus on sarcopenia^2,10,19,29^. 

 Although many studies that investigated nutritional status are already available in the literature, HGS assessment could contribute to the nutritional diagnosis of hospitalized patients. Hence, this study aimed to investigate the relationship between HGS and clinical outcomes in surgical ward inpatients. 

## METHODS

### Study design

 This was a cross-sectional study conducted in the surgical wards of a university hospital that serves a population in a large metropolitan region. The study was approved by the Institution’s Research Ethics Committee, and all subjects included in this investigation received detailed information about the study and signed the Informed Consent Form (ICF) (number 5.728.982). The Strengthening and Reporting of Observational Studies in Epidemiology (STROBE) guidelines were followed in this study^39^. 

### Subjects and inclusion criteria

 Adult patients admitted through the Unified Health System to the surgical wards of a hospital to treat different clinical and surgical diseases were selected. The inclusion criteria were patients who had complete information about the diagnosis, disease type, and complications duly recorded in their medical records; patients who did not have a terminal disease; patients who had been admitted for more than 24 hours; patients who could understand the information provided, with preserved motor and cognitive function; and patients who signed the ICF. Patients with edema or ascites, hospitalized in isolation, in a critical terminal condition, presenting with any type of dementia or Parkinson’s disease, and with fractures of the upper or lower limbs were excluded. To define the study sample, all patients monitored in the hospital surgical ward during the data collection period and who met all the eligibility criteria were included in the study (n=100 patients). 

### Data collection and variables

 Data were collected in the hospital, at the bedside, and retrieved from the medical records. All data were collected by the study investigators. The subjects were recruited upon hospital admission. The following variables were investigated:

1. Outcome variables: length of hospital stay, occurrence of complications, and hospital discharge or death.
2. Explanatory variables: type of disease, surgery, presence of comorbidities, gender, age, HGS, and nutritional indicators (body mass index (BMI) and subjective global assessment (SGA)).

HGS was assessed early after admission using a dynamometer at the bedside in the surgical ward, with the patient sitting. Patients were instructed to perform three maximum compressions with each hand, with brief pauses between measurements. Three measurements were taken with each hand, three with the dominant hand and three with the non-dominant hand. The mean of the three measurements was considered^2,13,24,31,34^. Low HGS values were obtained when the patient’s strength was <16 kg for women and <27 kg for men^10,13^. 

 To assess nutritional status, the subjective global assessment (SGA) instrument and BMI were used at the beginning of the hospitalization period. The SGA^11^ was assessed according to all the usual procedures, and the cutoff points were those recommended in the relevant literature. This instrument subjectively assesses the patient’s nutritional status based on weight loss, food consumption, and clinical and physical signs of malnutrition, which helped classify patients as: wellnourished, slightly malnourished, moderately malnourished, and seriously malnourished^11^. BMI was classified according to previously defined cutoff points^42^. 

### Statistical analysis

 The population characteristics were described using absolute frequency (n) and percentage (%) values for the categorical variables, and descriptive measures (mean, standard deviation, and median) for quantitative variables. The χ^2^ test or Fisher’s exact test was used to compare proportions, when necessary, and the Mann-Whitney test was applied to compare continuous measures between two groups. Spearman’s linear correlation coefficient was applied to assess the linear relationship between numerical variables. 

 Subsequently, to identify factors associated with complication outcomes, simple and multiple Cox regression analyses were used. To identify the variables that define hospital stay (in days), the Poisson regression model, which belongs to the special class of generalized linear models, was used. The significance level adopted for the statistical tests was 5%^8,35,37^. 

## RESULTS

 One hundred adult patients admitted to a surgical ward of a university hospital were evaluated; 63% (n=63) were male and 37% (n=37) female; the mean age of the subjects was 57.33±16.97 years (median=59.50 years). The most frequent diseases were vascular diseases (48%, n=48), neoplasms (20%, n=20), pulmonary diseases (9%, n=9), renal diseases (8%, n=8), orthopedic diseases (5%, n=5), digestive tract diseases (4%, n=4), and others (5%, n=5). Comorbidities were observed in 93% (n=93) of the patients, and 62% (n=62) had undergone surgical procedures. Regarding the indicators assessed, mean HGS was 22.78±10.67 (median=21.33) and BMI 26.28±5.85 kg/m^2^ (median=26.39 kg/m^2^). According to the SGA, 73% (n=73) of the patients were classified as well-nourished and 27% (n=27) as slightly malnourished. The length of hospital stay was 15.49±19.45 days (median=12 days), and complications were observed in 31% (n=31) of the patients treated. In this case series, one patient died. 

 In the linear correlation analysis using Spearman’s correlation coefficient, between age, HGS, and BMI with length of hospital stay as the clinical outcome, no significant linear correlations were found (age vs. length of hospital stay: - r=0.07367, p=0.4663, p>0.05; mean HGS vs. length of hospital stay: - r=-0.15878, p=0.1146, p>0.05; BMI vs. length of hospital stay: - r=-0.12207, p=0.2263, p>0.05). 

Table 1 shows the comparison of variables between genders: female patients showed lower mean (or median) HGS values than male patients (p<0.0001) and a higher complication rate (p=0.0425, p<0.05). 

**Table 1:** Descriptive analysis and comparison of variables between genders (N=100).

Variables studied	Category	Women (n=37)	Men (n=63)	Total (n=100)	p-value
Age (years)	Mean±SD	55.81±14.62	58.22±18.26	57.33±16.97	0.3855*
	Median	58.0	61.0	59.50	
Total length of hospital stay (days)	Mean±SD	14.19±10.48	16.25±23.21	15.49±19.45	0.5085*
	Median	13.0	10.0	12.0	
Average handgrip strength	Mean±SD	14.28±5.54	27.78±9.77	22.78±10.67	<0.0001*
	Median	13.83	27.0	21.33	
Body mass index (Kg/m^2^)	Mean±SD	26.78±6.33	25.98±5.57	26.28±5.85	0.2747*
	Median	27.46	25.92	26.39	
Complications (%)	No	21 (56.8)	48 (76.2)	69 (69.0)	0.0425†
	Yes	16 (43.2)	15 (23.8)	31 (31.0)	
Surgery (%)	No	12 (32.4)	26 (41.3)	38 (38.0)	0.3794†
	Yes	25 (67.6)	37 (58.7)	62 (62.0)	
Comorbidities (%)	No	2 (5.4)	5 (7.9)	7 (7.0)	1.0000‡
	Yes	35 (94.6)	58 (92.1)	93 (93.0)	
SGA (%)	Well nourished	26 (70.3)	47 (74.6)	73 (73.0)	0.6375†
	Slightly malnourished	11 (29.7)	16 (25.4)	27 (27.0)	

*Mann-Whitney test; †χ^2^ test; ‡Fisher’s exact test.

SD: standard deviation; SGA: subjective global assessment.

Table 2 shows the comparison of variables between the presence or absence of complications, with a significant difference only for the nutritional status classified by the SGA. There was a higher percentage of well-nourished patients among those who suffered complications (p=0.0333, p<0.05). There was no statistical difference in mean HGS regarding the occurrence or absence of complications. 

**Table 2:** Descriptive analysis and comparison of variables between the occurrence or not of complications (n=100).

Variables studied	Category	Without complications (n=69)	With complications (n=31)	Total (n=100)	p-value
Age (years)	Mean±SD	58.49±17.01	54.74±16.86	57.33±16.97	0.3650*
	Median	61.00	57.00	59.50	
Total length of hospital stay (days)	Mean±SD	14.01±12.01	18.77±30.08	15.49±19.45	0.5284*
	Median	12.00	13.00	12.00	
Average handgrip strength	Mean±SD	23.67±10.84	20.82±10.16	22.78±10.67	0.2078*
	Median	22.33	19.33	21.33	
Body mass index (Kg/m^2^)	Mean±SD	26.61±6.03	25.52±5.44	26.28±5.85	0.6123*
	Mean±SD	26.50	25.85	26.39	
Diagnosis (%)	Digestive tract disease	4 (5.8)	0 (0.0)	4 (4.0)	0.1136†
	Kidney/Urologic Disease	4 (5.8)	5 (16.1)	9 (9.0)	
	Vascular disease	33 (47.8)	15 (48.4)	48 (48.0)	
	Orthopedic disease	2 (2.9)	3 (9.7)	5 (5.0)	
	Lung disease	5 (7.2)	4 (12.9)	9 (9.0)	
	Oncological disease	16 (23.2)	4 (12.9)	20 (20.0)	
	Other diseases	5 (7.2)	0 (0.0)	5 (5.0)	
Surgery (%)	No	26 (37.7)	12 (38.7)	38 (38.0)	0.9219‡
	Yes	43 (62.3)	19 (61.3)	62 (62.0)	
Comorbidities (%)	No	5 (7.2)	2 (6.5)	7 (7.0)	1.0000†
	Yes	64 (92.8)	29 (93.5)	93 (93.0)	
SGA (%)	Well nourished	46 (66.7)	27 (87.1)	73 (73.0)	0.0333‡
	Slightly malnourished	23 (33.3)	4 (12.9)	27 (27.0)	

*Mann-Whitney test; †Fisher’s exact test; ‡χ^2^ test.

SD: standard deviation; SGA: subjective global assessment.

 When comparing the study variables between patients who underwent surgical procedures or not, there was a significant difference only in the presence of comorbidities. All patients who did not undergo surgery had some comorbidity (p=0.0422, p<0.05). 

Table 3 illustrates the comparison of the variables studied between the presence or absence of comorbidities. A significant difference was observed in relation to age (p=0.0462, p<0.05), mean HGS (p=0.0201, p<0.05), and diagnosis (p=0.0188, p<0.05). A higher mean (or median) age, lower mean HGS, and a higher rate of vascular disease, lung disease, and neoplasms were observed in patients with some comorbidity. 

**Table 3:** Descriptive analysis and comparison of variables between the presence or absence of comorbidities (n=100).

Variables studied	Category	Without comorbidity (n=7)	With comorbidity (n=93)	Total (n=100)	p-value
Age (years)	Mean±SD	42.71±18.66	58.43±16.42	57.33±16.97	0.0462*
	Median	35.00	60.00	59.50	
Total length of hospital stay (days)	Mean±SD	11.57±8.81	15.78±20.02	15.49±19.45	0.6168*
	Median	8.00	12.00	12.00	
Average handgrip strength	Mean±SD	33.10±11.94	22.01±10.22	22.78±10.67	0.0201*
	Median	37.83	21.00	21.33	
Body mass index (Kg/m^2^)	Mean±SD	28.15±7.16	26.13±5.76	26.28±5.85	0.5167*
	Median	27.97	26.33	26.39	
Diagnosis (%)	Digestive tract disease	1 (14.3)	3 (3.2)	4 (4.0)	0.0188†
	Kidney/urological disease	1 (14.3)	8 (8.6)	9 (9.0)	
	Vascular disease	1 (14.3)	47 (50.5)	48 (48.0)	
	Orthopedic disease	2 (28.6)	3 (3.2)	5 (5.0)	
	Lung disease	0 (0.0)	9 (9.7)	9 (9.0)	
	Oncological disease	1 (14.3)	19 (20.4)	20 (20.0)	
	Other diseases	1 (14.3)	4 (4.3)	5 (5.0)	
SGA‡ (%)	Well nourished	5 (71.4)	68 (73.1)	73 (73.0)	1.0000†
	Slightly malnourished	2 (28.6)	25 (26.9)	27 (27.0)	

*Mann-Whitney test; †Fisher’s exact test; ‡χ^2^ test.

SD: standard deviation; SGA: subjective global assessment.

 When comparing the study variables between the classifications of nutritional status by SGA, the patients classified as slightly malnourished were significantly characterized by a lower BMI (p=0.0052, p<0.05) and a higher neoplasm and lung disease rate. (p=0.0057, p<0.05). 

Table 4 illustrates the factors for complications analyzed by simple and multiple Cox regression. It was found that none of the variables assessed was considered an associated factor for complications. 

**Table 4:** Study of complication factors using simple and multiple Cox regression analysis.

Variables studied	Category	p-value	RR	95%CI
Simple analysis				
Age		0.3940	0.991	0.971–1.012
Average HGS		0.3043	0.982	0.948–1.017
BMI		0.4701	0.977	0.917–1.041
Gender	Female x Male	0.0968	1.816	0.898–3.673
Comorbidities	Yes x No	0.9048	1.091	0.260–4.574
SGA	Slightly malnourished x Well nourished	0.0877	0.401	0.140–1.145
Diagnosis	-	0.7328	-	-

Multiple analysis. Stepwise selection process. No variables selected at the 5% level.

RR: relative risk; CI: confidence interval; HGS: handgrip strength; BMI: body mass index; SGA: subjective global assessment.

Table 5 illustrates the factors that influence the length of hospital stay using Poisson regression analysis. It was found that the variables that, together, best explain the length of hospital stay were BMI (p<0.0001, p<0.05); mean HGS (p<0.0001, p<0.05); gender (p<0.0001, p<0.05), and diagnosis. BMI and mean HGS were inversely associated with the length of hospital stay (the lower the values, the longer the length of hospital stay). Male gender and vascular or orthopedic disease, compared to other diseases, evidenced longer hospital stays. 

**Table 5:** Factors influencing the length of hospital stay using Poisson regression analysis.

Variables studied	Category	Estimated parameter	Standard error	p-value
Simple analysis				
Age		0.0073	0.0015	<0.0001
Average HGS		-0.0134	0.0025	<0.0001
BMI		-0.0365	0.0046	<0.0001
Gender	Female x Male	0.1359	0.0537	0.0114
Comorbidities	Yes x No	0.3105	0.1141	0.0065
SGA	Slightly malnourished x Well nourished	-0.0572	0.0580	0.3242
Diagnosis	Digestive tract disease x Other	-0.2877	0.2202	0.1914
	Kidney disease x Other	-0.4055	0.1826	0.0264
	Vascular disease x Other	0.5498	0.1388	<0.0001
	Orthopedic disease x Other	0.3494	0.1761	0.0472
	Lung disease x Other	0.2491	0.1614	0.1228
Multiple analysis - Stepwise selection process				
BMI		-0.029020	0.004952	<0.0001
HGS		-0.020432	0.003443	<0.0001
Gender	Female x Male	0.302456	0.069615	<0.0001
Diagnosis	Digestive tract disease x Other	-0.313966	0.223672	0.1604
	Kidney disease x Other	-0.122443	0.186030	0.5104
	Vascular disease x Other	0.523687	0.138869	0.0002
	Orthopedic disease x Other	0.481158	0.176980	0.0066
	Lung disease x Other	0.049643	0.162220	0.7596
	Oncological disease x Other	0.142872	0.148069	0.3346

HGS: handgrip strength; SGA: subjective global assessment; BMI: body mass index.

## DISCUSSION

 The literature has demonstrated that HGS is considered an indicator of general muscle strength^4,16,30,34,38^ in many clinical situations^18,24,27^ and can also be used to assist in the assessment of malnutrition, playing a relevant role in this diagnosis^27,31,36,40^. 

 In our study, our objective was to assess the relationship between HGS and the clinical outcomes in patients admitted to a surgical ward; no linear correlation was observed between HGS and length of hospital stay. However, when analyzed using the Poisson regression, the factors influencing the length of hospital stay were HGS, BMI, gender, and diagnosis. To be specific, patients with lower HGS and BMI values experienced longer hospital stays, as did male patients and patients with vascular or orthopedic diseases. A robust study conducted with 1,136 patients reported that HGS had predictive value for length of hospital stay and quality of life^26^; a retrospective study conducted with 108 surgical patients selected with criteria similar to those of our investigation, also indicated that muscle strength assessed by manual dynamometry was a strong predictor of length of hospital stay in the surgical hospital setting^24^. Another prospective study that explored the association of HGS with clinical outcomes and its accuracy in identifying malnutrition showed that reduced HGS was associated with increased length of hospital stay and risk of death but did not show accuracy for malnutrition^7^. Another interesting finding in our study was the low HGS values in the population assessed: median 13.83 kg for women and 27.0 kg for men (the values of <16 kg for women and <27 kg for men were considered reduced HGS)^10,13^. 

 Although we may consider that measuring HGS is a useful, objective, relatively easy, non-invasive tool that can be performed at the bedside, and was the subject of our study, our findings did not show an association between HGS and the occurrence of complications. This differs from other studies that described this relationship^17,20,28^. This measurement was also not a predictor of postoperative morbidity in major hepatobiliary surgery, although it predicted a longer hospital stay, as observed in another single-center, prospective study with 81 patients^5^. 

 An association was observed between lower HGS values and the presence of comorbidities. Other investigations have also reported associations between HGS and causes of cardiometabolic multimorbidities^22^. In our study, an association was also observed among older age, a higher percentage of vascular and pulmonary diseases, and neoplasms in patients with some comorbidities. 

 Although other aspects related to other indicators of body composition and their modifications were not investigated in the present study because they were not the object of our investigation, perhaps in future studies it would be interesting to explore the differences between these indicators and HGS and between genders. It would also be relevant to explore these relationships concerning the clinical outcomes generally observed during hospitalization. 

 Another aspect to consider is the use of BMI. It is a widely used indicator, despite some known limitations, such as not separating lean mass from fat mass. Also, in the hospital setting, it is not used alone when it is possible to weigh patients. In routine hospital practice, other screening and nutritional diagnosis tools are strongly recommended and performed, such as nutritional risk screening, the universal malnutrition screening tool, the mini nutritional assessment in elderly patients, and the SGA, which was also used in the present study, in addition to BMI^6,9,14,15,29,43^. 

 It is important to highlight that our main objective was to investigate the association between muscle strength demonstrated by HGS and clinical outcomes during hospitalization. Therefore, our study, whose objective was not to investigate HGS in relation to the evolution or stage of the disease, has the following limitation: the impossibility to analyze the strength stratified or not in relation to the stage of the disease evolution, even because this would not be possible due to the variation of the small sample size in some clinical situations. Therefore, we suggest further investigations addressing these aspects in future studies. 

## CONCLUSIONS

 HGS and BMI were inversely associated with the length of hospital stay. Male gender and vascular or orthopedic diseases, compared with other diseases, caused a longer hospital stay. 

## Data Availability

The datasets generated and/or analyzed during the current study are available from the corresponding author upon reasonable request.
